# ScotCap – A large observational cohort study

**DOI:** 10.1111/codi.16029

**Published:** 2022-01-03

**Authors:** Campbell MacLeod, Jemma Hudson, Michelle Brogan, Seonaidh Cotton, Shaun Treweek, Graeme MacLennan, Angus J. M. Watson

**Affiliations:** ^1^ Department of Surgery Raigmore Hospital Inverness UK; ^2^ Health Services Research Unit University of Aberdeen Aberdeen UK; ^3^ Digital Health and Care Innovation Centre Glasgow UK; ^4^ Health Services Research Unit The Centre for Healthcare Randomised Trials University of Aberdeen Aberdeen UK

**Keywords:** colon capsule endoscopy, colorectal polyps, faecal immunochemistry testing, innovation, lower gastrointestinal diagnostics

## Abstract

**Aim:**

The aim of this work was to evaluate the performance of colon capsule endoscopy (CCE) in a lower gastrointestinal diagnostic care pathway.

**Method:**

This large multicentre prospective clinical evaluation recruited symptomatic patients (patients requiring investigation of symptoms suggestive of colorectal pathology) and surveillance patients (patients due to undergo surveillance colonoscopy). Patients aged 18 years or over were invited to participate and undergo CCE by a secondary‐care clinician if they met the referral criteria for a colonoscopy. The primary outcome was the test completion rate (visualization of the whole colon and rectum). We also measured the need for further tests after CCE.

**Results:**

A total of 733 patients were invited to take part in this evaluation, with 509 patients undergoing CCE. Of these, 316 were symptomatic patients and 193 were surveillance patients. Two hundred and twenty‐eight of the 316 symptomatic patients (72%) and 137 of the 193 surveillance patients (71%) had a complete test. It was found that 118/316 (37%) of symptomatic patients required no further test following CCE, while 103/316 (33%) and 81/316 (26%) required a colonoscopy and flexible sigmoidoscopy, respectively. Fifty‐three of the 193 surveillance patients (28%) required no further test following CCE, while 104/193 (54%) and 30/193 (16%) required a colonoscopy and flexible sigmoidoscopy, respectively. No patient in this evaluation was diagnosed with colorectal cancer. Two patients experienced serious adverse events – one capsule retention with obstruction and one hospital admission with dehydration due to the bowel preparation.

**Conclusion:**

CCE is a safe, well‐tolerated diagnostic test which can reduce the proportion of patients requiring colonoscopy, but the test completion rate needs to be improved to match that of lower gastrointestinal endoscopy.

**What does this paper add to the literature?**

This clinical evaluation demonstrated that colon capsule endoscopy (CCE) can be safely introduced into a diagnostic care pathway as an alternative to colonoscopy, and reduces the need for colonoscopy. The completion rate for CCE is acceptable but still needs to be improved to reduce the number of flexible sigmoidoscopy procedures that are required following CCE.

## INTRODUCTION

Colonoscopy remains the ‘gold standard’ colonic investigation. It is the most commonly utilized method for investigating patients with symptoms suggestive of colorectal cancer. It facilitates both the diagnosis of, and therapy for, colorectal pathology [1]. Most colonoscopy is performed under sedation, but despite a drive to increase the quality of colonoscopy, 8%–20% of procedures will be incomplete and patients find the procedure uncomfortable [2, 3, 4]. The diagnostic yield of colonoscopy in the symptomatic population is low, with 46%–75% of patients having a normal examination [3, 5]. Colonoscopy has a low complication rate (a 4–5 in 10,000 chance of perforation) [3, 6].

In many healthcare systems, the demand for colonoscopy outstrips supply and there are concerns around both the sustainability of services and waiting times [7, 8]. Long waits for diagnostic tests, particularly where a cancer diagnosis is concerned, lead to increased patient anxiety [9]. Alternative investigations, such as computed tomographic colonography (CTC) are available, but these are generally reserved for frail patients who will not tolerate colonoscopy, and test capacity is limited. CTC exposes patients to ionizing radiation, but the risks in general are low [10]. Given these challenges, there is a need to consider alternative colonic investigations that are acceptable to patients, can create capacity within the diagnostic system and that facilitate the early detection of colorectal cancer.

**FIGURE 1:**
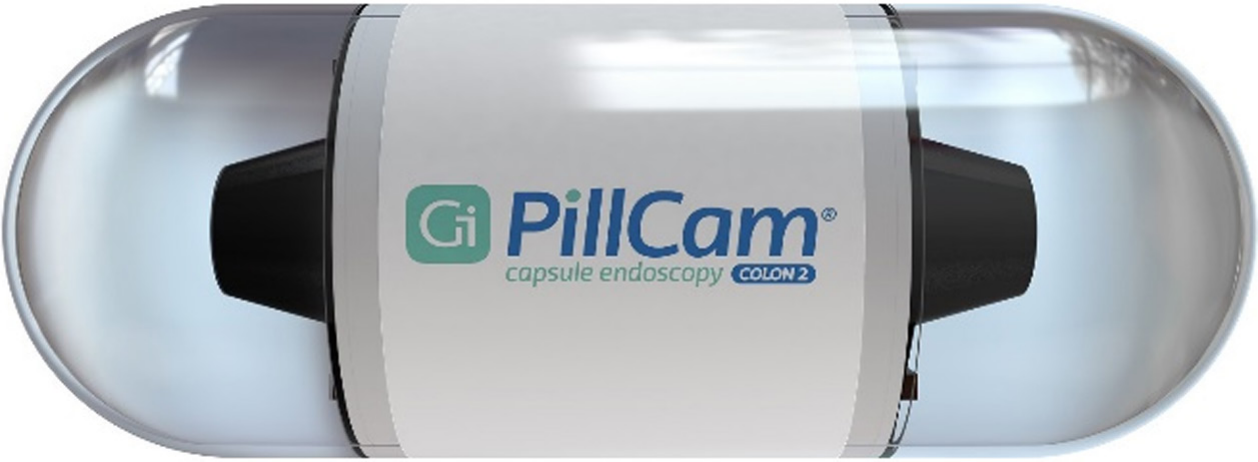
PillCam™ COLON 2 (copyright Medtronic)

Colon capsule endoscopy (CCE) is an alternative method for investigating the colon. The PillCam™ COLON 2 (Medtronic, UK) (Figure 1) is 32.3 mm long and 11.6 mm in diameter. It contains two cameras and it travels through the gastrointestinal system transmitting pictures wirelessly to a data recorder worn on a belt. Full bowel preparation with laxatives, equivalent to colonoscopy, is required to cleanse the colon before the procedure. However, in addition to these laxatives, ‘booster’ medicines are given after the capsule has been swallowed to promote colonic motility and capsule excretion. A successful CCE test is dependent on good bowel preparation and the capsule visualizing the whole of the colon and rectum. Rates for successful CCE tests vary between 54% and 88% depending on the bowel preparation regimen used and the population undergoing CCE [11].

All currently used diagnostic modalities are good at detecting colorectal cancer with acceptable false negative rates [12, 13, 14]. A recently published meta‐analysis and randomized control trial suggested that CCE is more sensitive than CTC for polyp detection [12, 15]. The principal advantages of CCE are that it can reduce the need for colonoscopy, it does not need specialist facilities to administer the test and it is noninvasive and painless. The main disadvantage of CCE (and CTC) is the potential need for follow‐up optical endoscopy for tissue diagnosis and therapy (polypectomy). Despite CCE having equivalent diagnostic utility to the other tests, adoption has been slow, with concerns about the high rate of follow‐up endoscopy and bowel preparation.

We therefore set out to perform a clinical evaluation of CCE within the National Health Service (NHS) in Scotland. Our aim was to assess the performance of CCE within a diagnostic care pathway and measure the need for additional diagnostic or therapeutic tests.

## METHOD

### Study design and participants

This large, multicentre, prospective clinical evaluation took place in three NHS Scotland health boards (Highland, Grampian and Western Isles). Patient recruitment was divided into two cohorts: symptomatic (patients requiring investigation of symptoms suggestive of colorectal pathology) and surveillance (patients being monitored because of an increased risk of development of colorectal cancer). Inclusion and exclusion criteria are described in Table 1.

**TABLE 1:** Inclusion/exclusion criteria

Patient type	Inclusion criteria	Exclusion criteria
All	Over 18 years of age Able to provide valid consent	Difficulty swallowing Indwelling electromedical device Insulin dependent diabetes mellitus History of small or large bowel strictures Pregnant woman Medically unfit to take full bowel preparation
Symptomatic	Referred from primary care with lower gastrointestinal symptoms and assessed as requiring a colonoscopy by a secondary‐care consultant	Predominant referral symptom diarrhoea or slow transit constipation FIT >400 µgHb/g Microcytic anaemia sole investigation reason
Surveillance	Due surveillance colonoscopy in the month before, during and month after recruitment period Personal or family history of colorectal cancer History of colonic polyposis HNPCC	Familial adenomatous polyposis Post‐endoscopic mucosal resection HNPCC with any polyps identified at previous colonoscopy More than five polyps at previous colonoscopy

Abbreviations: FIT, faecal immunochemical test; Hb, haemoglobin; HNPCC, hereditary nonpolyposis colorectal cancer.

Symptomatic patients were identified by hospital‐based clinicians vetting general practice (GP) referrals to the colorectal service. Symptomatic patients had undergone routine biochemistry and full blood count tests. A faecal immunochemical test (FIT) result was desired but not mandatory for symptomatic patients. Surveillance patients due to undergo colonoscopy between June 2019 and December 2019 were reviewed for inclusion.

All potential patients were invited to participate by introductory letter from the regional research and the endoscopy administration departments. Patients who declined CCE were treated according to standard care and offered colonoscopy. Demographic and baseline clinical data were collected by a data manager and recorded in the CASTOR Electronic Data Capture system, (a research database platform) for all patients undergoing CCE. The evaluation protocol is available online (https://www.dhi‐scotland.com/media/og5b0g3n/scotcap‐clinical‐evaluation‐protocol‐v2‐0‐final‐27‐08‐19‐1.pdf).

### Procedures

The capsule used in this evaluation was the PillCam™ COLON 2; the technical details and method use are detailed elsewhere [16]. Patients underwent CCE procedures in seven geographically convenient locations (four community healthcare centres and three district general hospitals) to minimize patient travel. The pretest dietary restriction, bowel preparation and booster regimen were consistent throughout the evaluation (Appendix 1). Procedures were supervised by trained nursing staff following a standardized protocol (Appendix 2). CCE reports were generated by consultant gastroenterologists based in Scotland (CCE reader) and returned electronically to the referring hospital clinician. An audit of 22 random reports was performed by an independent, experienced CCE reader, with low interobserver error (Appendix 3, Table C1).

The CCE reports detailed whether the examination was complete (if incomplete, the extent of colon visualized was reported), and included the bowel cleanliness rating according to the Boston bowel preparation scale by colonic segment (right colon, transverse colon, left colon including rectum) and the location, size and morphology of any colonic pathology [17]. CCE examinations were defined as complete if the capsule was excreted during its battery life or the anal cushions were visualized. The bowel preparation was deemed adequate if the rating was at least fair for all segments and the CCE reader judged the overall quality to be acceptable. Any relevant extracolonic (e.g. small bowel) findings were also reported.

The CCE report was reviewed by the referring clinician who decided if further management to investigate CCE findings, or incomplete tests, was needed. In general, polyps were examined by endoscopy as per European guidelines [18]. All patients for whom areas of the colon were inadequately visualized by the capsule due to an incomplete test or inadequate bowel preparation were referred for further investigation. Any further investigation (colonoscopy, flexible sigmoidoscopy or CTC) was carried out in line with local standard care. The CCE reports were made available to those doing further endoscopic procedures. The findings of the follow‐up investigations, and any associated pathology results, were collated by a data manager and recorded in CASTOR. The urgency rating (urgent suspected cancer, urgent, routine) of the follow‐up investigation request was also recorded. Adverse events related to the CCE procedures were recorded following a review of unplanned admissions in the patient’s electronic health record by a data manager and clinical researcher (CM). Adverse events associated with follow‐up investigations were not recorded.

### Outcomes

The primary outcome was the CCE test completion rate (excretion of the capsule within its battery life or visualization of the anal cushions). The secondary outcomes were rate of uptake of CCE, successful bowel preparation rate, findings from CCE (polyp, inflammation, colorectal cancer), the need for a further diagnostic bowel test (colonoscopy, flexible sigmoidoscopy, CTC) and findings and/or pathology found at further investigation (polyp, inflammation, colorectal cancer). In addition, we recorded the urgency and indication of follow‐up investigations, and the detection rate of colonic polyps and colorectal cancer in follow‐up tests.

### Statistical analysis

The primary outcome was determined as the proportion of complete CCE tests out of the total number of CCE procedures. The uptake of CCE was calculated as the proportion of patients who successfully swallowed the capsule out of those invited to participate in the evaluation. The number of patients requiring each follow‐up test was calculated as a sum of those who had undergone that test during the evaluation period plus those who were scheduled to undergo the test following the end of the evaluation period.

The requirement for follow‐up investigation was recorded from patients’ CCE reports by a clinical researcher (CM). Follow‐up tests were classified as ‘due to CCE findings’ if there were any findings reported by CCE necessitating endoscopy, regardless of whether the CCE examination was adequate. Investigations carried out solely due to an incomplete CCE and or inadequate bowel preparation were classified as ‘inadequate procedure’.

Polyp matching analysis was done for patients who underwent follow‐up endoscopic examination. Only colonic segments adequately visualized by CCE were considered for polyp matching purposes. For a polyp detected at CCE to be considered a true positive it had to match a polyp found at endoscopy located within the same or adjacent colonic segment (right, transverse and left colon including the rectum), and the size measured at CCE, plus or minus 50%, had to overlap with the size measured at endoscopy, plus or minus 50%. Polyps reported by CCE but not matched at endoscopy were regarded as false positives. Polyps detected by endoscopy but not reported by CCE were regarded as false negatives. To calculate the per‐polyp sensitivity, the number of true positive polyps was divided by the sum of true positive polyps and false negative polyps. Patients were regarded as true positives if they only had true positive polyps; if they had any false negative polyps the patient was regarded as a false negative. Using patient true positive and false negative data, per‐patient sensitivity was calculated. The chi‐square test was used to compare sensitivity results. The specificity of CCE in this evaluation was not calculated since not all patients underwent follow‐up endoscopy and we predicted that there would be only a small number of true negative patients.

We also calculated the number of colonoscopy appointments, classified by urgency rating, made available by the use of CCE. If a patient’s original referral was prioritized as urgent and they underwent CCE with no follow‐up required, one urgent colonoscopy appointment was ‘saved’. Thus, the demand for tests with different urgency could be calculated following CCE, taking into account the total number of follow‐up tests requested in different urgency categories.

## RESULTS

**FIGURE 2:**
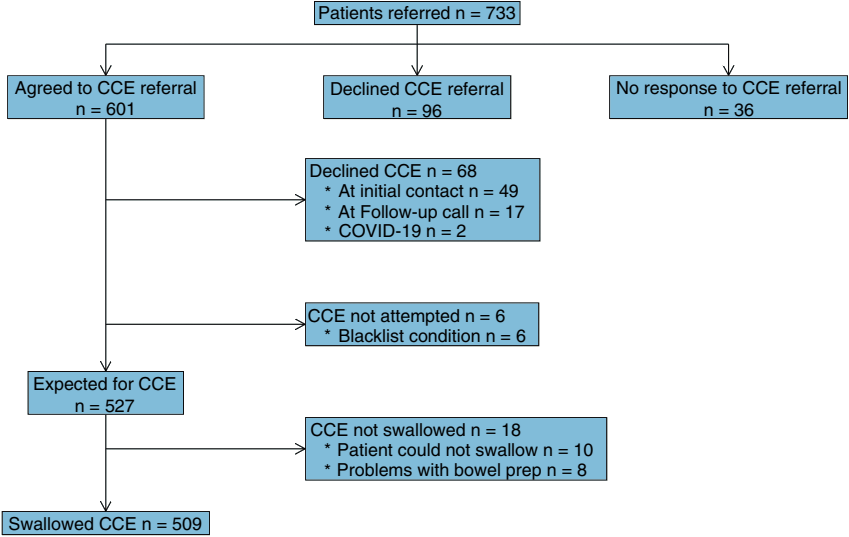
Participant flow (CCE, colon capsule endoscopy)

Between 10 June 2019 and 27 March 2020, 733 patients were invited to take part in this clinical evaluation with 509 subsequently undergoing CCE (Figure 2). On 3 December 2019, after interim analysis of the results, recruitment to the surveillance group was stopped because of concerns about the high rate of follow‐up tests compared with the symptomatic group. Recruitment to the evaluation was prematurely halted on 27 March 2020 in response to COVID‐19. Reasons for patients declining to take part are provided in Appendix 4 (Table D1). The clinical evaluation, and collection of follow‐up data, were terminated on 15 January 2021.

Baseline patient characteristics for each cohort that swallowed the CCE capsule are shown in Table 2. In total, 316 patients were symptomatic patients and 193 were surveillance patients. The majority of the symptomatic patients were female (57%) while the majority of surveillance patients were male (59%). A change in bowel habit was the most common referral symptom in the symptomatic group (66%). The presence of previous polyps was the most common reason for surveillance (52%). The mean full blood count haemoglobin for symptomatic patients was 141 g/l. The FIT data for symptomatic patients are available in Table 2. The mean time from patients being referred for CCE by secondary care to a report being available was 42 days, excluding patients who were unable to attend a first offered appointment. The referral priority for symptomatic patients is shown in Table 2. Three urgent suspected cancer (USC), 59 urgent and 41 routine colonoscopy appointments were required following CCE. Thus, 113/116 USC, 36/95 urgent and 64/105 routine colonoscopy slots were ‘saved’. Three USC, 54 urgent and 24 routine new flexible sigmoidoscopy appointments were required after CCE.

**TABLE 2:** Baseline characteristics

Symptomatic patients	*N* = 316
Mean age (years) (SD); *n*	58.9 (11.9); 316
Gender	
Female	179 (56.6)
Male	137 (43.4)
Urgency of referral	
Urgent suspected cancer	116 (36.7)
Urgent	95 (30.1)
Routine	105 (33.2)
Referral symptoms	
Change in bowel habit	209 (66.1)
Abdominal mass	132 (41.8)
Rectal bleeding	69 (21.8)
Positive FIT	63 (19.9)
Diarrhoea	60 (19.0)
Constipation	41 (13.0)
Weight loss	30 (9.5)
Microcytic anaemia	6 (1.9)
Other	12 (3.8)
Examination	
No mass felt	234 (74.1)
Mass felt	4 (1.3)
No examination	77 (24.4)
Missing	1 (0.3)
Mean Hb (g/L) (SD); *n*	141.3 (11.6); 230
FIT (µgHb/g)	
<10	113 (35.8)
10–399	85 (26.9)
≥400	5 (1.6)
Missing	113 (35.8)

Surveillance patients	*N* = 193
Mean age (years) (SD); *n*	62.9 (9.8); 193
Gender	
Female	80 (41.5)
Male	113 (58.5)
Reason for surveillance	
Previous polyps with fewer than polyps on previous colonoscopy	100 (51.8)
Colorectal cancer surgery follow‐up	35 (18.1)
Family history	23 (11.9)
HNPCC gene history (without family history of gastric cancer)	11 (5.7)
Other	24 (12.4)

Note

Values are *n* (per cent) unless otherwise stated.

Abbreviations: FIT, faecal immunochemical test; Hb, haemoglobin; HNPCC, hereditary nonpolyposis colorectal cancer.

The completion rate for the symptomatic patients was 228/316 (72%), and the proportion of patients with adequate bowel preparation was 251/316 (79%) (Table 3). Of those with a complete test, 208/228 (91%) had adequate bowel preparation. For surveillance patients, the completion rate and adequate bowel preparation rate was 137/193 (71%) and 127/193 (66%), respectively. Of those with a complete test, 110/137 (80%) had adequate bowel preparation. The left colon and rectum was the segment inadequately visualized for most patients with an incomplete test (Table 3). Five patients had an incomplete procedure due to retention of the capsule by the stomach with delayed excretion.

**TABLE 3:** Completion rates, bowel preparation rating and pathology detected

	Symptomatic (*N* = 316)	Surveillance (*N* = 193)
Complete test^a^	228 (72.2)	137 (71.1)
If incomplete complete to segment:		
Caecum	83/88 (94.3)	49/56 (87.5)
Right colon	81/88 (92.1)	46/56 (82.1)
Transverse colon	78/88 (88.6)	44/56 (78.6)
Left colon and rectum	69/88 (78.4)	31/56 (55.4)
Overall adequate bowel preparation^b^	251 (79.4)	127 (65.8)^c^
Adequate bowel preparation by segment		
Right colon	290 (91.2)	148 (76.7)
Transverse colon	284 (89.9)	150 (77.7)
Left colon and rectum	256 (81.0)	133 (68.9)

Results from those that required further test	Symptomatic (*N* = 193)	Surveillance (*N* = 138)
Polyp found	89 (46.1)	60 (43.5)
Largest polyp found		
≤6 mm	34 (38.2)	38 (63.3)
7–9 mm	33 (37.1)	13 (21.7)
≥10 mm	22 (24.7)	9 (15.0)
Number of polyps		
1–4	73 (82.0)	52 (86.7)
5–10	15 (16.9)	7 (11.7)
>10	1 (1.1)	1 (1.7)
Colorectal cancer	0 (0.0)	0 (0.0)
Inflammatory bowel disease	10 (5.2)	0 (0.0)
Diverticular disease^d^	1 (0.5)	0 (0.0)

Note

Values are *n* (per cent).

^a^ Defined as excretion of the capsule within its battery life or visualisation of the anal cushions.

^b^ Defined as adequate in all colonic segments and assessed as adequate overall by colon capsule endoscopy reader.

^c^ Missing for one participant.

^d^ Presence of diverticular disease was not routinely collected.

In the symptomatic group, 118/316 (37%) patients did not require any further test following CCE, while 103/316 (33%) required colonoscopy, 81/316(26%) required flexible sigmoidoscopy and 9/316 (3%) underwent CTC, with the remainder (5/316) either undergoing clinic review or requiring a laparotomy (Table 4). Of the 103 symptomatic patients requiring colonoscopy, 96 (92%) were due to CCE findings.

**TABLE 4:** Patient outcomes

	Symptomatic (*N* = 316)	Surveillance (*N* = 193)
No further test	118 (37.3)	53 (27.5)
Colonoscopy	103 (32.6)	104 (53.9)
Reason for test		
Due to CCE findings	95 (92.2)	82 (78.9)
Inadequate CCE	8 (7.8)	22 (21.2)
Complete CCE and inadequate bowl preparation	2 (1.9)	5 (4.8)
Incomplete CCE and inadequate bowl preparation	6 (5.8)	16 (15.4)
Incomplete CCE and adequate bowl preparation missing	0 (0.0)	1 (1.0)
Flexible sigmoidoscopy	81 (25.6)	30 (15.5)
Reason for test		
Due to CCE findings	36 (44.4)	17 (56.7)
Inadequate CCE	45 (55.6)	13 (43.3)
Incomplete CCE	40 (88.9)	11 (84.6)
Complete CCE and inadequate bowl preparation	1 (2.2)	0 (0.0)
Complete CCE and adequate bowel preparation	4 (8.9)	2 (15.4)
CTC	9 (2.9)	4 (2.1)
Reason for test		
Due to CCE findings	3 (33.3)	1 (25.0)
Inadequate CCE	6 (66.7)	3 (75.0)
Other^a^	5 (1.6)	2 (1.0)

Note

Values are *n* (per cent).

Abbreviations: CCE, colon capsule endoscopy; CTC, computed tomographic colonography.

^a^ Patients requiring review or laparotomy.

The results for the surveillance group showed that 53/193 (28%) of patients required no further test. Of those that did require a further test, 104/193 (54%) and 30/193 (16%) required colonoscopy and flexible sigmoidoscopy, respectively. Of the remaining patients in the surveillance group, 4/193 (2%) underwent CTC and 2/193 (1%) underwent clinic review. Of the patients who required flexible sigmoidoscopy procedures due to inadequate CCE, 40/45 (89%) and 11/13 (85%) were needed because of battery life exhaustion (rather than inadequate bowel preparation) in the symptomatic and surveillance group, respectively.

Of those that required a further test, 45% of symptomatic patients and 43% of surveillance patients had one or more polyps detected by CCE (Table 4). Of those patients with at least one polyp detected, the majority had polyps ≤6 mm (symptomatic 44%, surveillance 72%). No patients had colorectal cancer detected by CCE or any follow‐up test. Ten (5%) symptomatic patients had inflammatory bowel disease. Of those that required a further test, 46 patients had not undergone their planned follow‐up test by the end of the evaluation while 28 colonoscopy and 18 flexible sigmoidoscopy procedures were outstanding. The calculated sensitivity of CCE for the detection of polyps ≥6 mm and ≥10 mm on a per‐patient basis was 89.9% (95% CI 83.1–94.7) and 93.8% (95% CI 84.4–98.3), respectively. On a per‐polyp basis the calculated sensitivity was 91% (95% CI 86.3–93.9) for polyps ≥6 mm and 95.2% (95% CI 89.1–98.4) for polyps ≥10 mm. Table 5 shows the calculated number of true positive, false positive and false negative polyps for patients undergoing follow‐up endoscopy.

**TABLE 5:** Polyp matching analysis for patients undergoing endoscopy

	Polyp ≥6 mm	Polyp ≥10 mm
True positive polyps reported by CCE	230	99
False positive polyps reported by CCE	339	98
False negative polyps reported by CCE	24	5

Abbreviation: CCE, colon capsule endoscopy.

Two patients experienced serious adverse events. One developed bowel obstruction secondary to capsule retention due to undiagnosed Crohn’s disease. The patient underwent surgical management and made an uneventful recovery. The second patient developed dehydration due to the bowel preparation and boosters. This was managed with intravenous fluids. Two patients experienced nonserious adverse events. One patient developed a Mallory–Weiss tear due to vomiting after bowel preparation. One patient reported pain on excretion of the capsule. In addition, one patient experienced technical failure of the recorder, leading to them undergoing a colonoscopy, and one patient’s data recorder was temporarily misplaced due to a logistical error, resulting in them undergoing a CTC.

## DISCUSSION

In this multicentre prospective clinical evaluation, we found that 72% of symptomatic patients and 71% of surveillance patients had a complete CCE test. The CCE test completion rates need to be improved and must aspire to match those of optical colonoscopy (80%–92%). Whilst different ‘booster’ regimens have been trialled, substantially better completion rates will only be achieved when a capsule is equipped with a battery that enables the whole colon and rectum to be imaged for the majority of patients [11]. Forty of 45 flexible sigmoidoscopies for symptomatic patients and 11/13 for surveillance patients were performed because the left colon and/or rectum had not been visualized at CCE. A longer capsule battery life would have increased the completion rate, and probably reduced the number of flexible sigmoidoscopy procedures required.

For CCE to be considered as an alternative to colonoscopy or CTC, the proportion of patients that need a further test needs to be low. Our results show that symptomatic patients are more likely to require no further investigation than surveillance patients (37% vs. 28%) and are significantly less likely to require a follow‐up colonoscopy (33% vs. 54%). Pathology identified by CCE was the main reason for colonoscopy in the symptomatic group (92%), primarily for the therapy of diminutive (<6 mm) and intermediate (6–9 mm) polyps (38% and 37%, respectively). Diminutive polyps are more likely to be reported by CCE compared with optical colonoscopy and CTC due to the nature of the test [15, 19]. CTC reporting guidelines do not advocate the reporting of diminutive polyps [20]. Moreover, the chances of diminutive polyps progressing to a more sinister pathology or harbouring a cancer is 0% at 3 years [21]. Follow‐up endoscopy for these small polyps is still widely performed but is probably unnecessary.

The polyp detection rate of CCE is well established [12]. The per‐patient (93.8%) and per‐polyp (95.2%) sensitivity for polyps ≥10 mm in this evaluation is higher than in previously published research. A number of polyps ≥10 mm (98) reported by CCE were not found at follow‐up endoscopy. A minority may have been missed by the follow‐up test, but clinicians should be aware that polyps will be ‘over reported’ by CCE due to the nature of the test. No patients were diagnosed with colorectal cancer in this study and the published accuracy of CCE for detecting colorectal cancer is reassuring [12]. Furthermore, patients who have a lower risk of harbouring colorectal cancer are likely to be best suited to undergo CCE, as indicated by the low incidence of colorectal cancer in this evaluation. If CCE were to be used for patients on surveillance colonoscopy waiting lists (which postpandemic are currently very lengthy), although the need for follow‐up endoscopy is high, patients may value faster reassurance that they do not have bowel cancer.

Patient acceptance will be important to stakeholders when considering the introduction of new investigations. Qualitative research conducted in parallel to this study reported that patients found the procedure less invasive and painless compared with colonoscopy [22]. In addition, most patients (83%) indicated they would recommend CCE to others. The acceptance rate to participate in this service evaluation was high (69%), potentially reflecting the availability of CCE in geographically convenient locations or a desire to avoid colonoscopy. These findings are supported by other studies demonstrating CCE to be well‐tolerated by patients [23, 24, 25]. We must recognize, however, that colonoscopy will be preferred by some patients who decline CCE (24%) and it should continue to be discussed in any informed consent process.

Currently, there is a paucity of published cost‐effectiveness data comparing CCE with colonoscopy. An economic analysis using interim data from this evaluation has shown an additional per‐patient cost of GBP54 for a CCE diagnostic pathway, falling to GBP11 per patient in year 5, assuming wide‐scale adoption of the test [26]. Further cost benefit is likely to be accrued as the efficiency of CCE reading is improved with the introduction of machine learning algorithms to reduce the reading time [27]. The introduction of new and alternative colon capsules to the market will also reduce cost. Further cost‐effectiveness research is needed as the technology matures and completion rates improve.

One of the strengths of this evaluation is that it pragmatically evaluates the introduction of CCE into a standard clinical pathway. We captured clinician decision‐making based on CCE as a definitive test. Follow‐up procedures were prioritized by CCE findings and not by the urgency of the original GP referral. While the need for follow‐up endoscopy after CCE will be considered by some to be too high, the test did allow clinical teams to accurately triage the urgency of any follow‐up tests into a realistic timeframe. The overall effect of CCE, within a lower gastrointestinal diagnostic pathway, was to free up urgent colonoscopy appointments by reducing need or re‐triaging patients into a routine appointment.

There are some limitations to our study. Firstly, some patients (46/318) had not undergone their follow‐up tests by the end of the evaluation, with many delayed because of COVID‐19. However, based on their CCE findings, these patients were triaged to a low priority and are therefore unlikely to have clinically significant findings. We acknowledge this may also have influenced the calculated sensitivity of CCE in this evaluation as some patients may have findings missed by CCE. Secondly, this evaluation was carried out in three NHS Scotland health boards which have a significant rural population, which may reduce the generalizability of findings to other populations. To mitigate this, a strict protocol for carrying out the CCE procedures was used throughout the evaluation and broad inclusion criteria allowed a heterogeneous sample to be recruited. Rural provision of CCE for some patients in this evaluation reflects the potential flexibility in test delivery, and we believe it is not a barrier to reproducibility of outcomes in urban areas. Finally, adverse events were collected retrospectively, potentially reducing the number reported, particularly those that were not serious. Nevertheless, we are confident that the low rate of serious adverse events described is accurate. The rate of capsule retention in this study (0.2%) corresponds closely to that in a recent meta‐analysis (0.26%) [28].

## CONCLUSION

Most patients undergoing CCE have a complete test, but the completion rate requires improvement. In this evaluation, CCE reduced the proportion of symptomatic patients requiring colonoscopy, but was less beneficial for patients who required colonic surveillance. In addition, the costs of patients undergoing a second test were offset by the exclusion of colorectal cancer for many patients and improved triaging for endoscopic procedures, based on CCE findings. Given the current challenges experienced by endoscopy units, CCE is a safe and well‐tolerated alternative to colonoscopy that should be considered in the diagnostic lexicon.

## CONFLICT OF INTEREST

We declare no conflicts of interest related to this article.

## ETHICAL APPROVAL

The work was a service evaluation, therefore NHS research ethics committee and local research and development approvals were not required. A project board had oversight of the evaluation and met monthly.

## PATIENT CONSENT STATEMENT

Written consent was obtained from all patients participating in the evaluation.

## CLINICAL TRIAL REGISTRATION

The work was a service evaluation and therefore was not registered as a trial.

## PERMISSION TO REPRODUCE MATERIAL FROM OTHER SOURCES

Figure 1 is a picture of the PillCam COLON 2. Permission to use the picture has been given by Medtronic, UK.

## AUTHOR CONTRIBUTIONS

**Campbell MacLeod**: Study design, data collection, data analysis, manuscript writing; **Jemma Hudson**: Study design, study statistician, data analysis, manuscripy writing; **Michelle Brogan**: Study design; **Seonaidh Cotton**: Study design, data analysis, manuscript editing; **Shaun Treweek**: Study design, data analysis, manuscript editing; **Graeme MacLennan**: Study design, manuscript editing; **Angus J. M. Watson**: Study conception, study design, data analysis, writing and editing manuscript

## Data Availability

The data that support the findings of this study are available from the corresponding author upon reasonable request.

## APPENDIX 1

**Table:**

Bowel preparation and booster regime, and dietary recommendations		
Day	Preparation	Dietary recommendations
3 days before procedure	One sachet of Macrogol 3350 in 125 ml of water morning and evening	Normal diet
2 days before procedure	One sachet of Macrogol 3350 in 125 ml of water morning and evening	Low‐residue diet
1 day before procedure	Two litres of polyethylene glycol solution taken over 2 h in the evening	Clear liquid diet
Day of procedure	Two litres of polyethylene glycol solution taken over 2 h in the morning	Clear liquid diet
	Metoclopramide hydrochloride 10 mg tablet taken immediately after the colon capsule has been swallowed	Return to normal diet 6 h after capsule ingestion or once excreted
	One litre of sodium picosulfate solution taken as indicated by the three data recorder signals	
	One bisacodyl suppository (optional) to be used if the capsule has not been excreted 10 h after swallowing the capsule	

## APPENDIX 2

### COLON CAPSULE ENDOSOPY (CCE) PROCEDURE PROTOCOL

1. Patients agreeing to participate are screened by a nurse reviewing their electronic healthcare records.
2. The first telephone consultation is carried out by a nurse with the patient to explain the procedure in detail, confirm consent to continue with the procedure and arrange a test date.
3. The bowel preparation with instructions is sent to the patient’s home; regimen detailed in Appendix 1.
4. A second telephone consultation is carried out by a nurse with the patient to provide additional support for bowel preparation consumption.
5. The patient attends the CCE procedure, which is carried out by a trained nurse.
6. Preprocedure checks are carried out to ensure the patient is safe to continue with the procedure and that the bowel preparation has been adequate. If bowel cleanliness is inadequate, then one sachet of Picolax in 1 l of water is administered.
7. The belt and recorder are fitted, then the capsule is swallowed by the patient.
8. The booster medication is provided to the patient with further instructions.
9. The patient returns home to complete the procedure, returning the belt and recorder the following day.
10. The CCE recording is reported using the rapid reader software (Medtronic) by an NHS Scotland gastroenterologist trained in CCE reading.

## APPENDIX 3

**TABLE C1:** Colon capsule endoscopy report audit

Sample number	Original report				Auditor			
	Complete test	No. of polyps reported	Largest size of polyp (mm)	Clinical management based on report	Complete test	No. of polyps reported	Largest size of polyp (mm)	Clinical management based on report
1	Incomplete	3	<6	Colonoscopy	Incomplete	1	<6	Colonoscopy
2	Complete	4	8	Colonoscopy	Complete	4	15	Colonoscopy
3	Complete	5	<6	Nil	Complete	5	9	Nil
4	Incomplete	0	N/A	Flexible sigmoidoscopy	Incomplete	1	18	Colonoscopy
5	Complete	9	7	Colonoscopy	Complete	10	8	Colonoscopy
6	Complete	3	6	Nil	Complete	3	6	Nil
7	Complete	0	N/A	Nil	Complete	0	N/A	Nil
8	Complete	2	<6	Review	Complete	2	7	Review
9	Incomplete	Multiple	<6	Colonoscopy	Incomplete	>5	9	Colonoscopy
10	Complete	3	6	Nil	Complete	0	N/A	Nil
11	Incomplete	3	<6	Colonoscopy	Incomplete	4	9	Colonoscopy
12	Incomplete	2	<6	CTC	Incomplete	4	<6	CTC
13	Complete	0	N/A	Nil	Complete	0	N/A	Nil
14	Incomplete	2	8	Colonoscopy	Incomplete	1	<6	Colonoscopy
15	Incomplete	2	<6	Colonoscopy	Incomplete	3	8	Colonoscopy
16	Complete	2	7	Flexible sigmoidoscopy	Complete	1	7	Flexible sigmoidoscopy
17	Complete	2	<6	Nil	Complete	2	6	Nil
18	Complete	2	<6	Flexible sigmoidoscopy	Complete	2	7	Flexible sigmoidoscopy
19	Incomplete	0	N/A	Nil	Incomplete	0	N/A	Nil
20	Incomplete	2	<6	Flexible sigmoidoscopy	Incomplete	1	<6	Flexible sigmoidoscopy
21	Complete	0	N/A	Nil	Complete	3	6	Nil
22	Complete	2	10	Colonoscopy	Complete	2	10	Colonoscopy

Abbreviations: CTC, computed tomographic colonography; N/A, not applicable.

## APPENDIX 4

**TABLE D1:** Reasons for decline at referral and declining after agreeing to colon capsule endoscopy (CCE)

	*n* (%)
Declined at referral (*N* = 96)^a^	
No reason given	36 (37.5)
Prefer colonoscopy	31 (32.3)
Not interested/does not want CCE	12 (12.5)
Bowel prep (can't tolerate, doesn't want it)	5 (5.2)
Other medical conditions	5 (5.2)
Concerns about swallowing capsule/having capsule inside them	4 (4.2)
Time commitments (time off work, unable to do at present)	4 (4.2)
On advice	3 (3.1)
Out of area	2 (2.1)
Does not want CCE or colonoscopy	2 (2.1)
Travel	1 (1.0)
Other reasons	3 (3.1)
Declined after agreeing to the CCE referral (*N* = 68)^a^	
No reason given	26 (38.2)
Prefer colonoscopy	9 (13.2)
Other medical conditions	8 (11.8)
Time commitments (time off work, unable to do at present)	7 (10.3)
End of evaluation	6 (8.8)
Concerns about swallowing capsule/having capsule inside them	6 (8.8)
Bowel prep (can't tolerate, doesn't want it)	4 (5.9)
Travel	4 (5.9)
COVID‐19	2 (2.9)
Does not want CCE or colonoscopy	2 (2.9)
Not interested/does not want CCE	1 (1.5)
On advice	1 (1.5)
Out of area	1 (1.5)

^a^ Can be more than one reason.
